# Self-controlled feedback is effective if it is based on the learner’s performance: a replication and extension of Chiviacowsky and Wulf (2005)

**DOI:** 10.3389/fpsyg.2014.01325

**Published:** 2014-11-19

**Authors:** Michael J. Carter, Anthony N. Carlsen, Diane M. Ste-Marie

**Affiliations:** School of Human Kinetics, Faculty of Health Sciences, University of OttawaOttawa, ON, Canada

**Keywords:** motor learning, knowledge of results, self-control, learner-controlled, error estimation

## Abstract

The learning advantages of self-controlled feedback schedules compared to yoked schedules have been attributed to motivational influences and/or information processing activities with many researchers adopting the motivational perspective in recent years. Chiviacowsky and Wulf (2005) found that feedback decisions made before (Self-Before) or after a trial (Self-After) resulted in similar retention performance, but superior transfer performance resulted when the decision to receive feedback occurred after a trial. They suggested that the superior skill transfer of the Self-After group likely emerged from information processing activities such as error estimation. However, the lack of yoked groups and a measure of error estimation in their experimental design prevents conclusions being made regarding the underlying mechanisms of why self-controlled feedback schedules optimize learning. Here, we revisited Chiviacowsky and Wulf’s (2005) design to investigate the learning benefits of self-controlled feedback schedules. We replicated their Self-Before and Self-After groups, but added a Self-Both group that was able to request feedback before a trial, but could then change or stay with their original choice after the trial. Importantly, yoked groups were included for the three self-controlled groups to address the previously stated methodological limitation and error estimations were included to examine whether self-controlling feedback facilitates a more accurate error detection and correction mechanism. The Self-After and Self-Before groups demonstrated similar accuracy in physical performance and error estimation scores in retention and transfer, and both groups were significantly more accurate than the Self-Before group and their respective Yoked groups (*p*’s < 0.05). Further, the Self-Before group was not significantly different from their yoked counterparts (*p*’s > 0.05). We suggest these findings further indicate that informational factors associated with the processing of feedback for the development of one’s error detection and correction mechanism, rather than motivational processes are more critical for why self-controlled feedback schedules optimize motor learning.

## INTRODUCTION

A robust learning advantage for motor skill retention and transfer has consistently been demonstrated when learners are permitted control over their feedback schedule on a trial-to-trial basis (hereafter termed self-controlled) relative to externally imposed feedback schedules (hereafter termed yoked; see Wulf, 2007; Sanli et al., 2013 for reviews). In this context, feedback refers to information that may not normally be available to the learner [i.e., knowledge of results (KRs)], but can be provided by an external source such as a coach to augment naturally occurring movement-related feedback. The purpose of comparing motor performance on retention and transfer tests between groups with, and without control over their KR schedule is to confirm that any group differences in learning are attributable to having control over KR during practice, rather than a function of the frequency to which KR was provided or the amount of practice itself. Moreover, retention and transfer tests provide complimentary, yet different information regarding the characteristics of learning. Retention tests evaluate the relative permanence of one’s performance capability acquired during practice while transfer tests assess the generalizability or adaptability of what was learned in practice (Schmidt and Lee, 2011, p. 462; Kantak and Winstein, 2012).

Although the learning benefits of self-controlled KR schedules are well documented (see Wulf, 2007; Sanli et al., 2013 for reviews), no clear explanation of the mechanisms underlying the optimization of motor learning under these conditions exists. Currently, two explanations are predominantly used to account for the learning benefits of self-controlled KR schedules in the motor domain. According to the motivational (or psychological) explanation, a self-controlled KR schedule satisfies the basic psychological needs of autonomy and competence, as KR can be chosen for perceived successful trials, which results in higher levels of intrinsic motivation and subsequent learning (e.g., Chiviacowsky et al., 2012a; Ste-Marie et al., 2013; Chiviacowsky, 2014). Recently, many researchers investigating the underlying mechanisms for the learning advantages of self-controlled KR have largely favored this motivational perspective (e.g., Wulf et al., 2010; Chiviacowsky et al., 2012a,b,c; Ste-Marie et al., 2013; Chiviacowsky, 2014). In fact, Sanli et al. (2013) encouraged future investigations to enhance our understanding of self-controlled learning benefits from the motivational perspective via Self-determination theory (Ryan and Deci, 2000).

The alternative to the motivational view is the information processing perspective which suggests that the learning benefits of self-controlled KR are predominately driven by the learner’s ability to engage in *performance-dependent* KR strategies – which increases the relative value of the feedback received compared to yoked schedules which are not performance-dependent (e.g., Patterson and Carter, 2010; Hansen et al., 2011; Patterson et al., 2011). For example, some researchers have found that participants in self-controlled groups report a preference for requesting KR after perceived good trials (Chiviacowsky and Wulf, 2002; Patterson and Carter, 2010) while others have reported mixed results for the self-reported strategies used during practice (see both Patterson et al., 2011, 2013). More recently it has been shown that participants in a self-controlled KR group reported a strategy of requesting KR equally following perceived good and poor trials early in practice, but switched to requesting KR only after perceived good trials during the later stages of practice (Carter and Patterson, 2012). Together these findings highlight the performance-dependent nature of self-controlled KR schedules throughout the practice phase.

Although the motivational perspective has garnered much attention in recent years (see Wulf et al., 2010; Sanli et al., 2013 for discussions), there is a seemingly overlooked finding from a paper by Chiviacowsky and Wulf (2005). In that paper, the authors stated that informational processes associated with the processing of KR, rather than motivational processes, may be more critical for explaining why self-controlled KR schedules optimize motor learning. To elaborate, Chiviacowsky and Wulf (2005) investigated whether the temporal locus of the KR decision, made either *before* (Self-Before) or *after* (Self-After) motor execution, differentially impacted learning of a sequential timing task. It was argued that if the learning benefits of self-controlled KR were predominately related to motivational influences, then the timing of one’s KR decision should not affect motor learning as both groups would still be self-controlling their KR delivery. In contrast, if information-based factors related to the processing of KR (e.g., subjective performance evaluations) have a greater contribution to the learning benefits, then motor learning should depend on the timing of the KR decision (Chiviacowsky and Wulf, 2005). The authors found no significant group differences on a delayed retention test; however, the Self-After group was significantly more accurate than the Self-Before group on a delayed transfer test^1^. To account for these findings, error scores on KR versus no-KR trials during practice were examined and it was found that errors were significantly lower on KR trials compared to no-KR trials for both groups. This finding for the Self-After group replicated their earlier work (see Chiviacowsky and Wulf, 2002) but was an unexpected finding for the Self-Before group. As a result, Chiviacowsky and Wulf (2005, p. 46) suggested participants in the Self-Before group may have “tried harder after deciding they wanted feedback for a particular trial” so they would have a success experience once KR was provided. The authors further speculated that both groups “may have benefited from a motivational influence of self-control…[but] this factor alone cannot explain the learning advantages [on the transfer test] of the Self-After condition” (p. 46). It was therefore concluded that being able to make the KR decision after movement execution allowed learners to base their decision on their (estimated) performance (Chiviacowsky and Wulf, 2005); thus, resulting in informational benefits. Further, they argued that a more accurate error detection and correction mechanism may at least partially subserve the learning benefits of self-controlled KR schedules. The methodological limitation of no yoked groups in their experiment, however, does not allow for conclusions regarding the underlying mechanisms, whether motivational or informational, for why self-controlled KR learning advantages emerge. Their experimental design also did not include any measure associated with error detection and correction, and thus further research including an assessment of this mechanism as a critical factor for why self-controlled KR schedules optimize motor learning is warranted (see Carter and Patterson, 2012).

In the present experiment, we revisited the work of Chiviacowsky and Wulf (2005) and addressed their primary methodological limitation via the addition of yoked groups in order to investigate the involvement of motivational and informational processes to the learning advantages of self-controlled KR schedules. Our experiment also extends their work through two features: first, all participants were asked to make performance estimations after each trial during the retention and transfer tests of the experiment to examine the hypothesis that error estimation processes have an important role in self-controlled KR learning benefits (Chiviacowsky and Wulf, 2005; Carter and Patterson, 2012). Second, we included a novel self-controlled group that was provided the option to request KR *before* a trial but could then *change or stay* with their original decision *after* a trial. Thus, three self-controlled KR groups were compared: one that completed their KR decision *before* a trial (hereafter Self-Before), one that made the decision *after* a trial (hereafter Self-After), and one in which learners decided both *before* and *after* a trial (hereafter Self-Both). The Self-Both group was added to test a potential positive additive effect of motivational and informational factors related to self-controlling one’s KR delivery. More specifically, and in line with ideas presented by Chiviacowsky and Wulf (2005), this group may be motivated to try harder to have a “success experience” after choosing KR for an upcoming trial; however, they could then also engage in subjective performance evaluations to determine whether KR would in fact be valuable for that particular trial. Thus, these learners would have the assumed advantages associated with both motivational and informational processes for both motor skill retention and transfer, and may therefore benefit more than those learners who only gain a single assumed advantage (i.e., the Self-Before group [primarily motivational] and the Self-After group [primarily informational]).

Of secondary interest was to examine whether participants in the self-controlled groups would exhibit decreased error on trials where KR was requested (e.g., Chiviacowsky and Wulf, 2002, 2005) as a strategy to have “success experiences” to protect perceptions of competence (Chiviacowsky et al., 2012a; Chiviacowsky, 2014). It was predicted that if motor learning is optimized through self-controlled KR schedules due to a combination of motivational influences and information processing activities, then the Self-Both group should demonstrate superior learning relative to the Self-Before and Self-After groups. Moreover, differences in skill transfer were expected between the Self-Before and the Self-After group (e.g., Chiviacowsky and Wulf, 2005). Consistent with the existing literature, all self-controlled feedback groups were expected to demonstrate enhanced motor learning and a more accurate error detection and correction mechanism relative to their respective yoked counterparts.

## MATERIALS AND METHODS

### PARTICIPANTS

Forty-eight volunteers (30 Female, 18 Male; *M*_age_ = 21.35, SD = 1.12 years) with no self-reported sensory or motor dysfunctions participated in the experiment after giving written informed consent. The first 24 participants were randomly assigned to one of the self-controlled groups while the last 24 participants were randomly assigned to one of the yoked groups. This resulted in six equal-sized (*n* = 8) groups: Self-Before, Self-After, Self-Both, Yoked-Before, Yoked-After, and Yoked-Both. The experiment was approved and conducted in accordance with the ethical guidelines set by the Health Sciences and Science Research Ethics Board at the University of Ottawa.

### TASK AND APPARATUS

**FIGURE 1:**
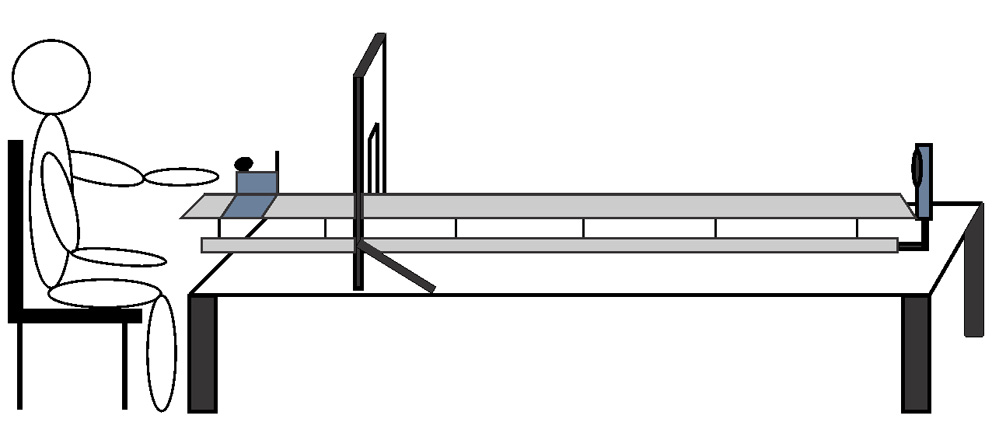
**A schematic representation of the slider apparatus**.

Participants were informed the goal of the motor task was to propel a low-friction slider along a horizontal rail such that it would stop at a target distance of 133 cm (see **Figure 1**). Thus, the task was similar to a force production task in which the participant is required to learn the correct amount of force to exert to reach the goal distance. Participants were in a seated position and grasped the handle of the slider (12.1 cm × 17.1 cm [L × H]; 455 g) with a transverse palmar grip using their non-dominant hand. Hand dominance was determined using the Edinburgh Handedness Inventory (Oldfield, 1971). The horizontal rail was 261.6 cm in length with the first 50 cm of the rail defined as a pre-response area. A wooden barrier (78.7 cm × 45.7 cm) was located 50 cm from the start of the horizontal rail and participants were informed the wooden barrier represented the 0 cm position relative to the target distance. The barrier contained an opening slightly larger than the slider to allow unobstructed travel along the rail. Moreover, all participants wore opaque goggles during all experimental phases to ensure they did not look through the small opening to see the end location of the slider. This was to ensure participants would rely on proprioceptive information to learn the task rather than visual information. A Vernier Motion Detector 2 (ultrasound frequency of 50 kHz with an accuracy of ±2 mm within a range of 0.5–6 m) was mounted to the end of the horizontal rail and was used to detect the end position of the slider relative to the zero position (i.e., wooden barrier) during all experimental phases. The Vernier Motion Detector 2 was connected to a Vernier LabPro^®^ that collected and transmitted the position data of the slider on each trial, and was calibrated each day prior to testing. The Vernier Motion Detector 2 and the Vernier LabPro^®^ were controlled using a customized LabVIEW program (National Instruments Inc.) which also controlled the timing of all experimental stimuli and stored all data for oﬄine analysis.

### EXPERIMENTAL DESIGN AND PROCEDURE

The self-controlled groups were informed they would have control over their KR schedule, but with the restriction they *must* request KR on three of 10 trials in each practice block (consistent with Chiviacowsky and Wulf, 2005). KR requests were restricted to ensure that any potential learning differences between the three self-controlled groups could not be due to differences in the relative frequency of KR during practice. The self-controlled groups were also instructed to only request KR when necessary because they would eventually be required to perform the task without KR. Participants in the self-controlled groups were further informed about when they would be asked to make their KR decision in accordance with their respective experimental group (i.e., informed they would be asked before, after, or both before and after a trial). Participants in the yoked groups received the identical KR schedule to that of a self-controlled counterpart in each practice block. Participants in the yoked groups were informed that KR would be provided according to a pre-determined schedule and that the researcher would indicate whether KR would or would not be provided either before, after, or both before and after a trial.

**FIGURE 2:**
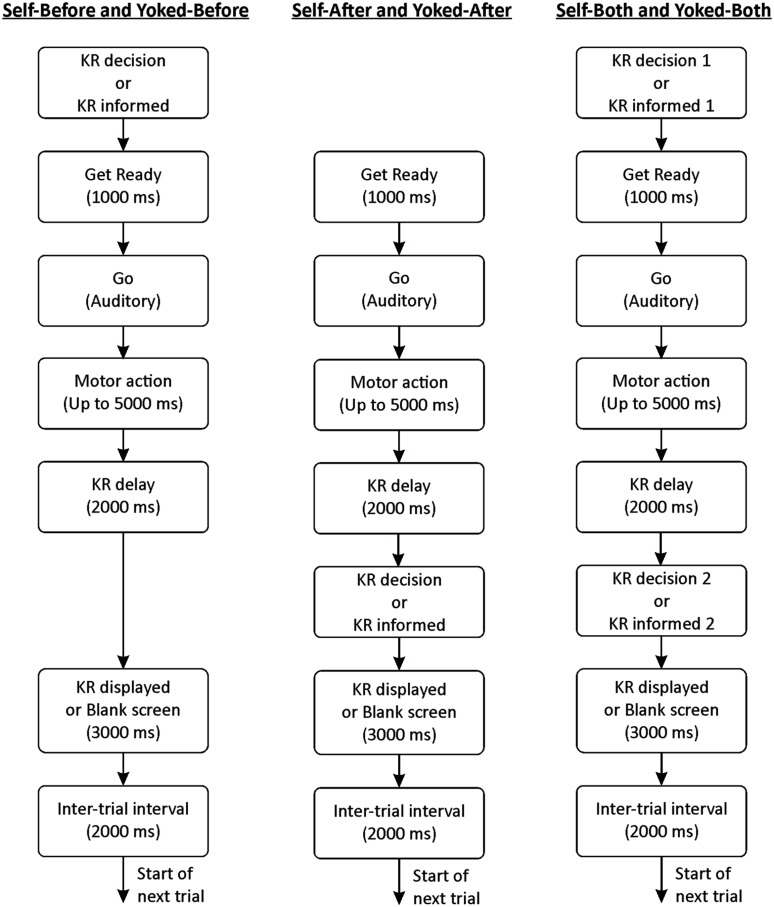
**Temporal events in a typical practice trial as a function of experimental group**.

Consistent with the methods of Chiviacowsky and Wulf (2005), testing was completed over two consecutive days, with the practice phase on Day 1 and the retention and transfer tests on Day 2. The practice phase began with participants reading through a series of instructions outlining the goal of the motor task and their respective experimental group. During the practice phase, all participants completed 60 trials (six blocks of 10 trials) with a relative KR frequency of 30% (i.e., three KR trials per 10 trial block). For all experimental trials, participants were allowed up to 5 s to complete their motor action. On KR trials during practice, participants removed their opaque goggles to view KR that was displayed on a 19-inch LCD monitor for 3 s. The KR display consisted of the target distance (133 cm), the distance of their motor response (e.g., 123), and their constant error score (e.g., –10 cm). The timeline of a typical experimental trial is illustrated in **Figure 2**. The retention and the transfer tests consisted of 10 no-KR trials with the transfer test requiring participants to adapt to a new target distance (165 cm). To further test the notion that an enhanced ability to detect and correct errors may underlie the learning benefits of self-controlled KR schedules, all participants were asked to estimate their perceived outcome of each motor response during retention and transfer.

### STATISTICAL ANALYSES

Absolute error (AE) scores were calculated for all phases of the experiment. To examine the development of the error detection and correction mechanism, the absolute difference (AD) between the participant’s estimated outcome and their actual outcome was calculated for the retention and transfer tests. These dependent measures were used to index changes in motor performance and learning and were analyzed using analysis of variance (ANOVA) procedures described below. An alpha level of ≤0.05 was considered significant and where appropriate, partial eta squared ($\eta_{p}^{2}$) is reported to provide an estimate of effect size. To decompose significant effects, *post hoc* tests were administered using Tukey’s HSD and/or Holm–Bonferroni procedures. In cases where sphericity was violated, Greenhouse–Geisser adjusted *p* values are reported.

## RESULTS

### PRACTICE

#### Absolute error

**FIGURE 3:**
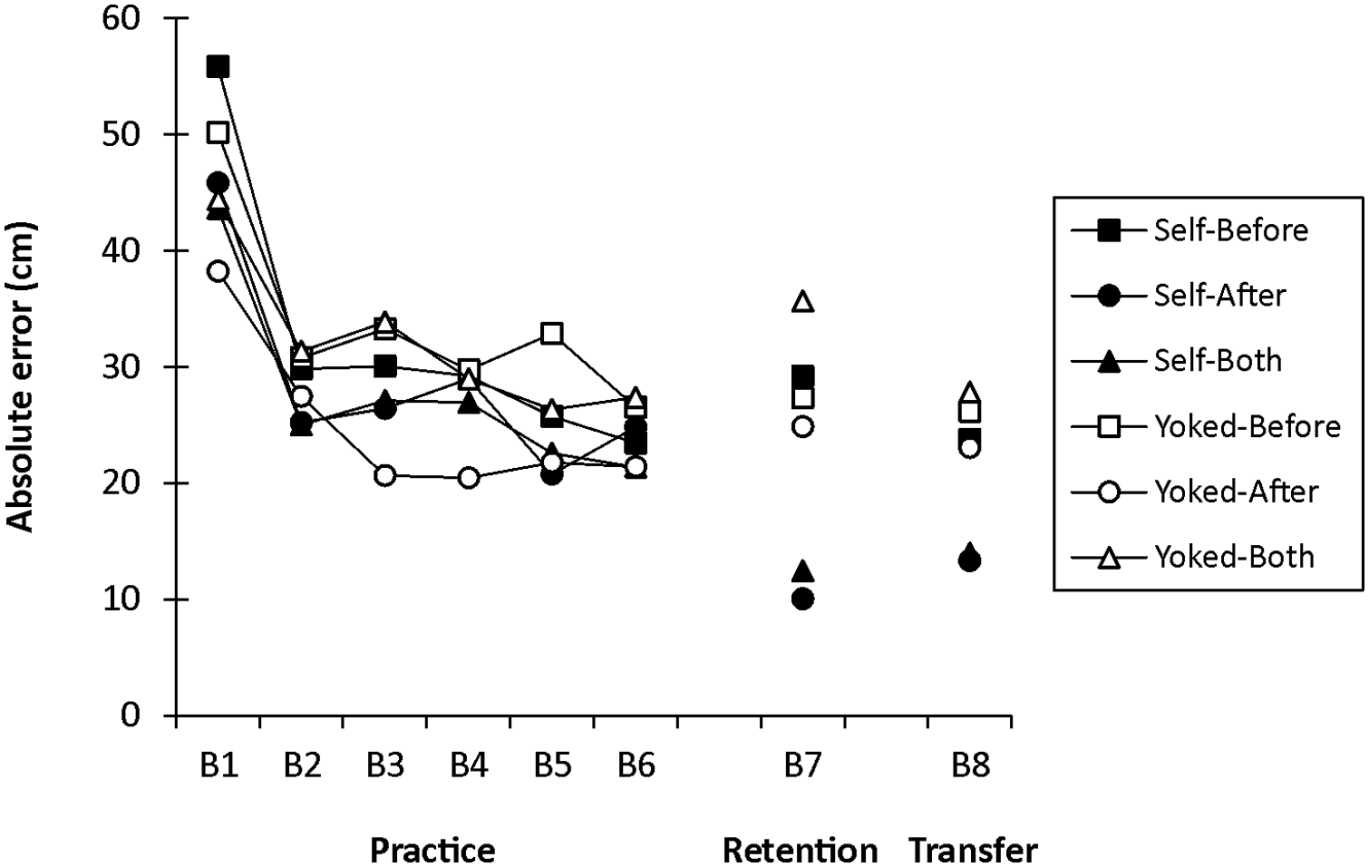
**Mean absolute error (cm) as a function of choice, decision time, and block (B1–B6 = practice; B7 = 24-h retention test; B8 = 24-h transfer test)**. Each block includes 10 trials and feedback was only available for Blocks 1–6.

Absolute error scores (cm) for practice are shown in **Figure 3** (B1–B6) and were analyzed using a 2 (Choice: Self, Yoked) × 3 (Decision: Before, After, Both) × 6 (Block) mixed-model ANOVA with repeated measures on Block. All groups showed a reduction in AE across practice blocks, which was supported by a significant main effect, *F*(5,210) = 39.20, *p* < 0.001, $\eta_{p}^{2}$ = 0.48. A significant main effect of Decision was also found, *F*(2,42) = 4.50, *p* = 0.017, $\eta_{p}^{2}$ = 0.18, with *post hoc* analyses showing that independent of choice only the After groups were significantly more accurate during practice than the Before groups. All other comparisons failed to reach statistical significance (*p* > 0.05).

#### AE on KR versus no-KR trials

Consistent with the analysis used by Chiviacowsky and Wulf (2005), mean AE was calculated on KR and no-KR trials for the first and second half of the practice phase (see **Table 1**) and analyzed using a 2 (Choice) × 3 (Decision) × 2 (Type: KR, no-KR) × 2 (Half: First, Second) mixed-model ANOVA. A significant Choice × Type interaction was found, *F*(1,42) = 5.80, *p*= 0.021, $\eta_{p}^{2}$ = 0.12, and *post hoc* testing showed the Yoked groups performed with lower AE on no-KR relative to KR trials, whereas no differences were noted on KR versus no-KR trials for the self-controlled groups. There was also a significant Half × Type interaction, *F*(1,42) = 14.96, *p*< 0.001, $\eta_{p}^{2}$ = 0.26, with *post hoc* tests revealing that AE on KR trials for the first half of practice was significantly greater than AE on KR trials for the second half of practice and no-KR trials for both halves of practice. Moreover, AE on no-KR trials was significantly lower in the second half relative to the first half of practice.

**Table 1:** Mean AE (±SE) scores (cm) on KR trials and no-KR trials during the first and second half of practice for each experimental group.

	First half (trials 1–30)		Second half (trials 31–60)	
Group	KR	no-KR	KR	no-KR
Self-Before	42.41 (3.64)	**36.92 (2.72)**	26.37 (3.72)	**26.05 (2.20)**
Self-After	**31.22 (2.98)**	32.21 (3.68)	**20.50 (2.89)**	24.96 (2.77)
Self-Both	36.82 (2.69)	**30.62 (2.29)**	**20.26 (1.07)**	26.83 (3.00)	
Yoked-Before	44.39 (5.72)	**35.36 (2.57)**	**28.57 (4.14)**	30.20 (3.80)
Yoked-After	31.94 (2.81)	**27.44 (1.85)**	**19.34 (2.74)**	22.01 (1.23)
Yoked-Both	40.52 (3.66)	**34.82 (2.65)**	31.85 (3.09)	**25.73 (1.65)**

*For each practice half, the boldfaced and underlined numbers highlight the trial type (either KR or no-KR) for which performance was more accurate during practice*.

#### KR scheduling within practice blocks

Although the relative frequency of KR was controlled (i.e., three KR trials per block) it was possible that participants could distribute their KR trials differently within the 10 trials which in turn could produce differential effects on performance and learning. To rule this out, we determined the frequency distribution for which trials (1–10) the three self-controlled groups self-scheduled their KR collapsed across practice (see **Table 2**). Both the Self-Before and the Self-Both groups used their KR requests predominantly on trials 1, 2, and 3 whereas the Self-After group predominantly requested KR on trials 1, 3, and 4. Thus, all three self-controlled groups appear to have favored more of a massed schedule when using their KR requests wherein KR was requested primarily for early trials rather than later trials in a practice block.

**Table 2:** The amount KR was requested for trials 1–10 collapsed across practice for the three self-controlled groups (and its percentage of total KR request opportunities in parentheses).

	Trial number										
Group	1	2	3	4	5	6	7	8	9	10
Self-Before	**26 (18.1%)**	**22 (15.3%)**	**20 (13.9%)**	19 (13.2%)	13 (9%)	8 (5.6%)	12 (8.3%)	13 (9%)	6 (4.2%)	5 (3.5%)
Self-After	**32 (22.2%)**	16 (11.1%)	**24 (16.7%)**	**21 (14.6%)**	17 (11.8%)	17 (11.8%)	9 (6.3%)	4 (2.8%)	3 (2.1%)	1 (0.7%)
Self-Both	**34 (23.6%)**	**26 (18.1%)**	**25 (17.4%)**	10 (6.9%)	6 (4.2%)	15 (10.4%)	6 (4.2%)	8 (5.6%)	8 (5.6%)	6 (4.2%)

*The boldfaced and underlined numbers highlight the three trial numbers within a block for which KR was most often requested*.

### RETENTION AND TRANSFER

#### Absolute error

Absolute error scores for the retention and transfer tests are shown in **Figure 3** (B7, B8 respectively) and were analyzed using separate 2 (Choice) × 3 (Decision) two-way ANOVAs. In retention, the main effects for Choice and Decision were superseded by a significant Choice × Decision interaction, *F*(2,42) = 7.13, *p* = 0.002, $\eta_{p}^{2}$ = 0.25. *Post hoc* comparisons revealed that although the Self-After (*M* = 10.04, SE = 1.89) and Self-Both (*M* = 12.45, SE = 3.58) groups did not differ significantly, they were both significantly more accurate in retention than the Self-Before group (*M* = 29.18, SE = 4.08). In addition, Self versus respective Yoked comparisons revealed: (1) both the Self-After and Self-Both groups had significantly less AE than their Yoked counterparts (Yoked-After: *M* = 24.87, SE = 3.04; Yoked-Both: *M* = 35.66, SE = 3.55); and (2) the Self-Before and the Yoked-Before (*M* = 27.35, SE = 3.66) groups did not differ significantly.

Similar to retention, the main effects for Choice and Decision during the transfer test were superseded by a significant Choice × Decision interaction, *F*(2,42) = 3.46, *p*= 0.041, $\eta_{p}^{2}$ = 0.14. *Post hoc* analyses revealed the following: (1) the Self-After (*M* = 13.31, SE = 0.98) and Self-Both (*M* = 13.96, SE = 2.38) groups performed with significantly less AE than the Self-Before group (*M* = 23.77, SE = 1.85) and their respective Yoked groups (Yoked-After: *M* = 23.06, SE = 1.47; Yoked-Both: *M* = 27.80, SE = 2.87); and (2) the Self-Before and the Yoked-Before (*M* = 26.18, SE = 2.92) groups were not significantly different.

#### Absolute difference

**FIGURE 4:**
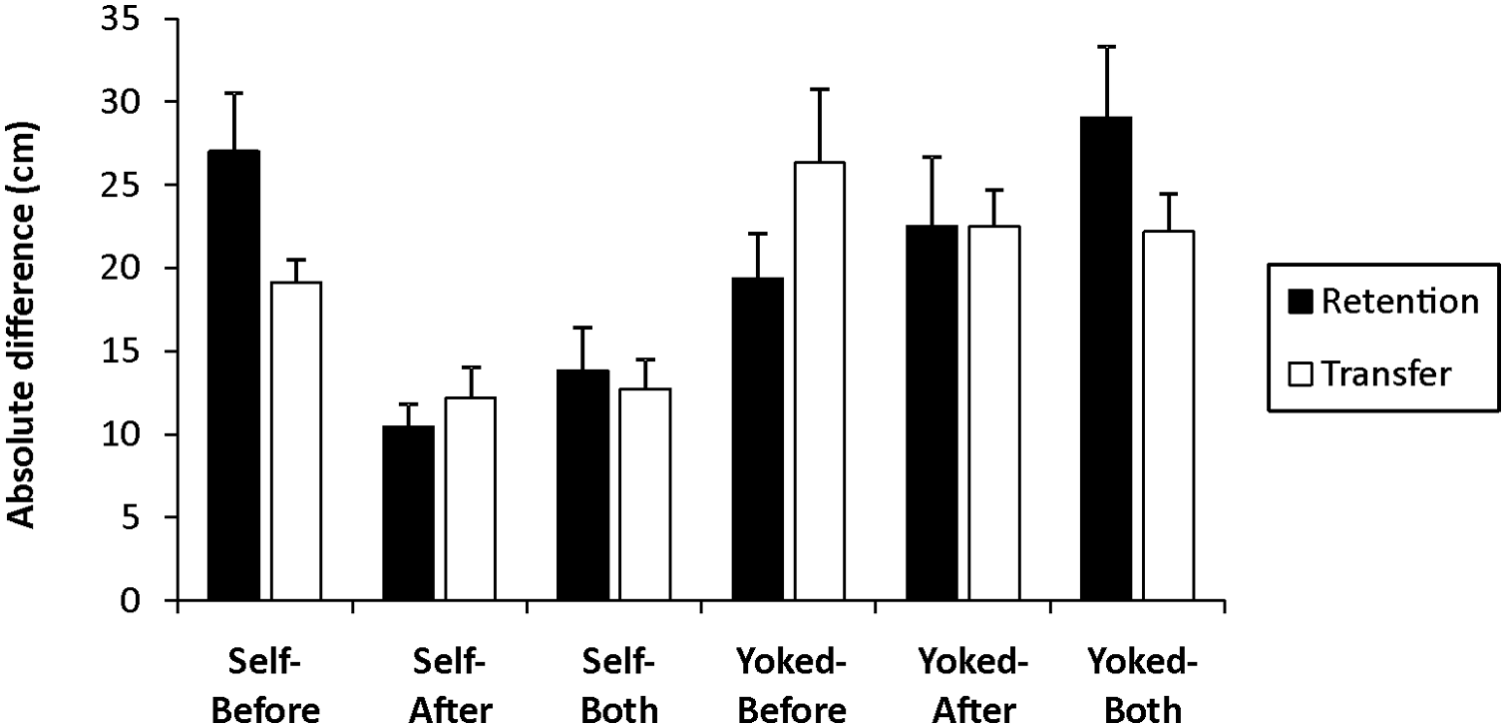
**Mean (±SE) absolute difference (cm) as a function of choice, decision time, and test (Retention = black; Transfer = white).** Each test consisted of 10 trials without feedback. Absolute difference was calculated by subtracting the participant’s estimated outcome from their actual outcome.

Absolute difference scores for retention and transfer for each group are displayed in **Figure 4** and were analyzed in separate 2 (Choice) × 3 (Decision) two-way ANOVAs. The main effects for Choice and Decision in retention were superseded by a significant interaction, *F*(2,42) = 7.19, *p*= 0.002, $\eta_{p}^{2}$ = 0.26, which revealed that both the Self-After (*M* = 10.44, SE = 1.40) and the Self-Both (*M* = 13.80, SE = 2.59) groups were significantly more accurate in their subjective performance evaluations than the Self-Before group (*M* = 27.01, SE = 3.52). Moreover, the Self-After and the Self-Both groups did not differ significantly but both were significantly more accurate than their respective yoked counterparts (Yoked-After: *M* = 22.51, SE = 4.16; Yoked-Both: *M* = 29.02, SE = 4.32) whereas the Self-Before group was not statistically different than the Yoked-Before group (*M* = 19.37, SE = 2.67).

In transfer, only the main effect for Choice was significant, *F*(1,42) = 19.29, *p*< 0.001, $\eta_{p}^{2}$ = 0.31, with *post hoc* analysis indicating the self-controlled groups (*M* = 14.68, SE = 1.14) were significantly more accurate than the Yoked groups (*M* = 23.67, SE = 1.76). As can be seen in **Figure 4**, this significant main effect for Choice appears to be predominantly driven by the estimations of the Self-After and Self-Both groups. Due to the more accurate estimations in retention and our main interest in comparisons amongst the three self-controlled groups, we conducted a separate one-way ANOVA to examine differences in subjective performance estimations between the three self-controlled groups. The analysis revealed a significant main effect, *F*(2,21) = 5.24, *p*= 0.014, $\eta_{p}^{2}$ = 0.33, where *post hoc* tests revealed no differences between the Self-After (*M* = 12.19, SE = 1.86) and Self-Both (*M* = 12.73, SE = 1.79) groups, but both were significantly more accurate than the Self-Before group (*M* = 19.12, SE, 1.34).

## DISCUSSION

While it has consistently been shown that self-controlled KR schedules enhance motor learning relative to yoked schedules (see Wulf, 2007; Sanli et al., 2013 for reviews), most studies have primarily focused on evaluating the effectiveness of self-controlled KR rather than investigating the relative contributions of motivational and informational processes underlying these learning benefits. The present experiment revisited Chiviacowsky and Wulf (2005) with three important modifications: (1) the inclusion of yoked groups (a noted limitation in their design), (2) a dependent measure related to error estimation to examine possible informational benefits of self-controlled KR schedules, and (3) the creation of a novel self-controlled group to test a potential positive additive effect of the posited motivational and informational factors associated with self-controlled KR (see Wulf, 2007; Sanli et al., 2013 for reviews). For this latter purpose, we included three self-controlled groups that were assumed to be reflective of varying levels of informational and motivational contributions. It was thought that the Self-Before group, whose decision to receive KR was restricted to *before* each trial would gain primarily motivational advantages. In comparison, it was presumed the Self-After group, whose decision was made *after* each trial, would gain advantages due to informational processes. Advantages due to both motivational and informational processes were expected for our novel Self-Both group that made a KR decision *before* a trial, but were given the option to change or stay with their original choice *after* the trial. Our inclusion of yoked groups for all self-controlled groups was a strength of the present experiment and were necessary to understand the proposed contributions of motivational and informational processes to the learning benefits of self-controlled KR schedules.

The present data did not support the hypothesis of a positive additive effect of motivational and informational processes on learning under self-controlled KR conditions. Instead, the critical factor for increased learning appears to be the opportunity to decide *after* motor execution whether they want KR. This is because both the Self-After and the Self-Both groups significantly outperformed the Self-Before group in retention and transfer, yet did not differ significantly from one another. Moreover, the Self-After and the Self-Both groups demonstrated significantly more accurate retention and transfer performance compared to their respective yoked groups. In contrast, the Self-Before group showed similar performance to the Yoked-Before group. Thus, simply having control over one’s KR schedule prior to motor execution did not result in a learning benefit compared to a corresponding yoked group. These results therefore replicate and extend the work of Chiviacowsky and Wulf (2005) and highlight that self-controlled learning benefits *depend* on the option of making the KR decision after completing one’s motor response.

It is difficult for an explanation based on motivational influences to reconcile why the timing of the KR decision would modulate the learning benefits of self-controlled KR schedules. According to a purely motivational explanation, no differences in motor learning would be expected as all three groups are assumed to have received the same degree of autonomy regarding their choice over when to receive or not receive KR because all self-controlled participants had three KR requests per practice block. Although one limitation of the current study is that no autonomy or motivation measure was collected regarding choice over one’s KR schedule^2^, support for our assumption comes from recent work by Ste-Marie et al. (2013) who found learning benefits of self-controlled feedback schedules despite participants in the self-controlled and yoked groups not differing significantly in their perceived choice (i.e., autonomy) or interest/enjoyment (i.e., motivation) throughout practice. Further challenges to the motivational perspective have also emerged in recent years with the finding that limiting or decreasing the amount of self-control opportunities (i.e., a less autonomy supportive context) compared to a traditional self-control group (i.e., a more autonomy supportive environment due to unlimited request opportunities) does not hinder learning (Patterson et al., 2011) and can also lead to superior learning (Hansen et al., 2011). Such findings (see Carter and Patterson, 2012; Patterson et al., 2013 for other examples) suggest that although an autonomy supportive environment afforded by self-controlled KR may contribute to increased motivation and subsequent learning benefits, a more critical factor may be the additional information processing activities engaged during practice.

A question that remains concerns which critical information processing activities are not (sufficiently) engaged when the learner completes their KR decision before, rather than after a motor response? The data from the current experiment support Chiviacowsky and Wulf’s (2005) proposition that *error estimation* is a critical process underlying why self-controlled KR schedules optimize learning when the decision is made after motor execution. This conclusion is based on the superior retention and transfer data (both AE and AD) shown in the present experiment and in recent work by Carter and Patterson (2012). The motor behavior-memory framework (Kantak and Winstein, 2012), which highlights the importance of encoding processes (e.g., error estimation) during practice for the development of an accurate memory representation would suggest that practicing under conditions where the KR decision is made before a trial (or KR is imposed on the learner without any choice) does not preclude the learner from engaging in error estimation processes. Instead, it seems plausible to suggest any encoding and subsequent learning benefits that can be derived from error estimation are diminished in these practice conditions. For example, KR may be requested (or provided) on a trial where it only provides information that is redundant with response-produced feedback (e.g., Magill et al., 1991; Buekers et al., 1992; Hale and Franks, 1998; Kernodle et al., 2001). Alternatively, KR may not be requested (or provided) for a trial where it would have provided valuable information to strengthen one’s memory representation. In fact, research has shown that error estimation during practice is only effective for learning if it is followed by the presentation of KR for that trial (Guadagnoli and Kohl, 2001). In the current experiment, both groups that had the ability to request KR after a trial showed superior performance during retention and transfer. We therefore suggest that having the option to request KR after motor performance allows the learner to request KR only when a comparison between perceived and actual error would maximize the informational value of KR received (i.e., reduce uncertainty because information is transmitted; Fitts and Posner, 1967; Marteniuk, 1976; Guadagnoli and Lee, 2004). An examination of the Self-Both group’s behavior regarding staying or changing their original decision provides support for this contention. Although it was more common for participants to stay with their original choice (108 times for “yes” and 293 times for “no”), there were 30 occasions wherein the KR decision changed from “no” to “yes” and 13 times that it changed from “yes” to “no.” Despite these differences, the underlying and crucial similarity of each instance is that no matter the outcome of the second decision, the participants were always able to base their final KR decision following motor execution. This would optimize encoding processes related to the development and strengthening of a more accurate error detection and correction mechanism, which, in the absence of continued motor training, would facilitate the retrieval of a more permanent and adaptable memory representation as measured using retention and transfer tests, respectively (Kantak and Winstein, 2012).

It is interesting to note that during and at the end of practice all groups demonstrated a comparable level of skill proficiency (see **Figure 2**). This is consistent with the extant self-controlled feedback literature (e.g., Chiviacowsky and Wulf, 2002; Patterson and Carter, 2010; Ste-Marie et al., 2013) and highlights the fact these robust advantages do not seem to manifest until a period of no practice has occurred. Two possible explanations may account for this phenomenon in the present experiment and the self-controlled KR literature in general: motor memory consolidation (Robertson et al., 2004; Robertson, 2009; Kantak and Winstein, 2012) and transfer-appropriate processing (Morris et al., 1977; Bransford et al., 1979; Lee, 1988).

Consolidation is a set of post-practice (i.e., oﬄine), time-dependent processes that enhance the memory representation that was encoded during practice (Kantak and Winstein, 2012), with these oﬄine improvements thought to be sleep-dependent (e.g., Walker et al., 2002, 2003; Walker and Stickgold, 2004). The present data suggest that making the KR decision after a trial resulted in oﬄine improvements (i.e., lower error following the retention interval for the Self-After and the Self-Both groups). Unfortunately, the design of our experiment does not allow a true assessment of oﬄine learning as a comparison between Block 6 and retention is problematic as these trials were completed under different levels of the independent variable. The inclusion of an immediate retention test would be required to gain better insight into the degree of forgetting and enhancement (i.e., oﬄine learning; see Lin et al., 2008; Goh et al., 2012 for examples of this analysis) associated with different self-controlled and yoked KR schedules. Nevertheless, the current data provides initial support that the learning benefits of self-controlled KR schedules (if the decision is made after a trial) are potentially related to enhanced consolidation processes over the retention interval (Walker et al., 2002, 2003; Robertson et al., 2004; Walker and Stickgold, 2004).

Alternatively, the delayed benefits of self-controlled KR schedules may relate to memory retrieval processes. According to the framework of transfer-appropriate processing, learning is optimized when the processing activities promoted by the practice condition resemble the processing activities that are required by the learning tests. Because retention and transfer tests are typically performed without the provision of KR, participants must rely on their error detection and correction mechanism to evaluate and modulate their motor performance on these tests. The encoding processes associated with strengthening one’s ability to detect and correct errors appears to be encouraged when the option to request KR after motor performance is provided. Thus, the superior retention and transfer performance of the Self-After and the Self-Both groups relative to the other experimental groups may also relate to transfer-appropriate processing activities (see Lee, 1988 for a discussion specific to the motor learning domain).

A minor limitation to note in the design of the experiment is the small timing variation in the KR-delay intervals between the groups (see **Figure 2**). The Self-Before group had a fixed 2000 ms before KR would or would not be displayed, while the other two self-controlled groups had 2000 ms plus the decision time concerning KR delivery. This marginally greater delay between movement completion and KR delivery could be argued to have allowed the engagement of additional error estimation processes to benefit learning that were not available in the fixed 2000 ms interval. However, we specifically adopted a 2000 ms KR delay interval based on past research revealing that error estimation processes are engaged immediately following a movement (McGuigan, 1959; McGuigan et al., 1960; Dyal et al., 1965; Dyal, 1966; Newell, 1976; see Salmoni et al., 1984; Swinnen, 1988; Swinnen et al., 1990 for in-depth discussions). Therefore, any error estimation processes would be expected to have occurred very quickly following movement completion and well within the fixed 2000 ms KR delay interval used for all groups. As well, although we did not measure KR decision time between the groups, these decisions were made very quickly by participants. Given the above information, we remain confident the learning differences between the Self-After and the Self-Both groups relative to the Self-Before group are related to optimization of the informational value of the KR received rather than to any marginal increases in time between movement completion and KR delivery.

Of final interest was examining whether differences in movement accuracy would emerge between trials where KR was or was not requested. Chiviacowsky and Wulf (2005) reported lower error scores on KR trials compared to no-KR trials (see also Chiviacowsky and Wulf, 2002). This led to the notion that participants with control over KR may favor receiving KR on more accurate trials as a way to protect perceptions of competence; which in turn enhances learning through motivational factors (e.g., Chiviacowsky et al., 2012a). Although our data showed a trend for decreased error on KR versus no-KR trials during the second half of practice (for the Self-After, Self-Both, Yoked-Before, and Yoked-After groups), these differences were not statistically significant (see **Table 1**). As a result, the superior learning of the Self-After and Self-Both groups is difficult to attribute to these participants requesting KR predominantly after more accurate trials as a way to protect perceptions of competence. Chiviacowsky (2014) recently showed that when perceptions of competence associated with KR after successful trials was controlled for between a self-controlled and a yoked group, learning advantages for the self-controlled group still emerged. This further suggests that motivational factors are at best, a minimal contributing mechanism for the learning benefits of self-controlled KR schedules.

Inspection of our data also revealed that participants had greater error on KR trials relative to no-KR trials early in practice; however, later in practice this trend switched (see **Table 1**). This may have been a function of how participants chose to distribute their KR requests within practice blocks (see **Table 2**) as all groups seemed to favor asking for KR on the early trials in a block. This behavioral data, along with subjective KR strategy reports in Carter and Patterson (2012) seem to support an informational role of KR requests early in practice, presumably to help calibrate performance toward the task goal (e.g., Salmoni et al., 1984). In the later blocks of practice, KR may be requested in more of a reinforcement role. That is, KR on more accurate trials may strengthen or help consolidate the learners’ memory association between the predicted and actual motor outcomes (Patterson and Carter, 2010).

In conclusion, we investigated whether a positive additive effect of motivational and informational factors was a viable explanation for the learning advantages associated with self-controlled KR schedules. While the results did not support this additive effect, the robust self-controlled learning advantages did emerge for those participants who had the option to request KR following their performance. We suggest that these advantages were primarily due to encoding advantages associated with the informational value of KR for error estimation processes. The current data support the conclusions of Chiviacowsky and Wulf (2005, p. 45) that “self-control *per se*…and perhaps associated increases in motivation…is not the determining factor for the benefits of self-controlled KR”. As such, we recommend further investigation into the associated encoding advantages gained from self-controlled KR schedules as the key underlying mechanism for self-controlled motor learning benefits.

## Conflict of Interest Statement

The authors declare that the research was conducted in the absence of any commercial or financial relationships that could be construed as a potential conflict of interest.

## Footnotes

1 The authors argued that transfer performance is a more sensitive measure of learning than retention performance. However, both retention and transfer performance should be viewed as important indices of learning as they provide different, yet complimentary information regarding characteristics of learning (i.e., permanence and adaptability of a memory representation for skill, respectively).

2 It is important to point out a concern regarding the use of subjective measures for variables such as autonomy and/or motivation in the motor learning domain. Researchers have primarily adopted components of the Intrinsic Motivation Inventory (IMI; McAuley et al., 1989) to examine these psychological variables (e.g., subscales of interest/enjoyment, perceived choice, perceived competence, and effort/importance). However, a major limitation of using the subscales of the IMI that has not been addressed is that questions are phrased in terms of the task (e.g., taken from Ste-Marie et al., 2013: I enjoyed doing this double mini activity very much) rather than in terms of having control over the variable of interest (e.g., I enjoyed having control over my feedback schedule). Lastly, researchers interested in examining a causal relationship between motor learning and these psychological constructs would require significantly larger sample sizes in order to run the appropriate structural equation modeling analyses.
